# A Sporadic Case of Disseminated Fusobacterium Causing Pylephlebitis and Intracranial and Hepatic Abscesses in a Healthy Young Patient

**DOI:** 10.7759/cureus.9229

**Published:** 2020-07-16

**Authors:** Biswaraj Tharu, Bisrat Nigussie, Daniel Woredekal, Fuad I Abaleka, Mahalet Gizaw

**Affiliations:** 1 Internal Medicine, Western Reserve Health Education/NEOMED, Youngstown, USA; 2 Internal Medicine, Richmond University Medical Center, Staten Island, USA; 3 Department of Medicine, Trumbull Regional Medical Center, Youngstown, USA; 4 Neurology, Mount Sinai Hospital, New York, USA

**Keywords:** pylephlebitis, intracranial abscess, hepatic abscess

## Abstract

Fusobacterium species is known for being a causative agent for Lemierre’s syndrome, which is characterized by thrombophlebitis of the jugular vein. However, Fusobacterium species causing Lemierre’s variant gastrointestinal syndrome has only been reported in case reports. We present a case of Fusobacterium causing hepatic vein pylephlebitis, multiple brain abscesses, and hepatic abscess. To the best of our knowledge, there have only been four other case reports of Fusobacterium causing hepatic abscesses with associated septic pylephlebitis in the literature.

## Introduction

Fusobacterium species causing bacteremia is a very rare occurrence, with a reported incidence of only 0.38-0.55/100,000 per annum [1-3]. Fusobacterium species are gram-negative anaerobic bacilli that are commonly known for causing Lemierre’s syndrome, especially in healthy young adults and children. The subspecies that is associated with Lemierre’s syndrome is Fusobacterium necrophorum (F. necrophorum). In Lemierre’s disease, the bacteria infect oropharynx and invade into the internal jugular vein, resulting in thrombophlebitis. Fusobacterium has also been reported in case reports to cause intra-abdominal infections and septic thrombophlebitis [4,5]. In this report, we present a case of Fusobacterium nucleatum (F. nucleatum) with simultaneous liver, brain and, and hepatic pylephlebitis.

## Case presentation

**Figure 1:**
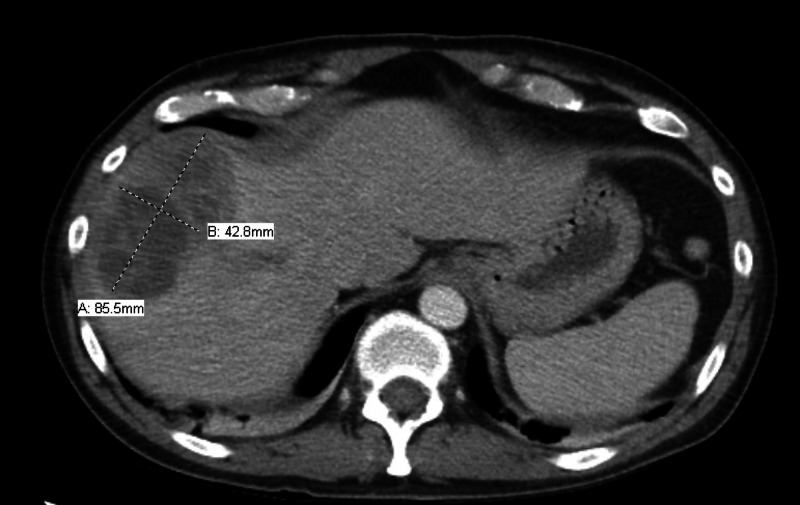
Initial CT scan of abdomen The cross mark on the left upper side shows hepatic abscess CT: computed tomography

**Figure 2:**
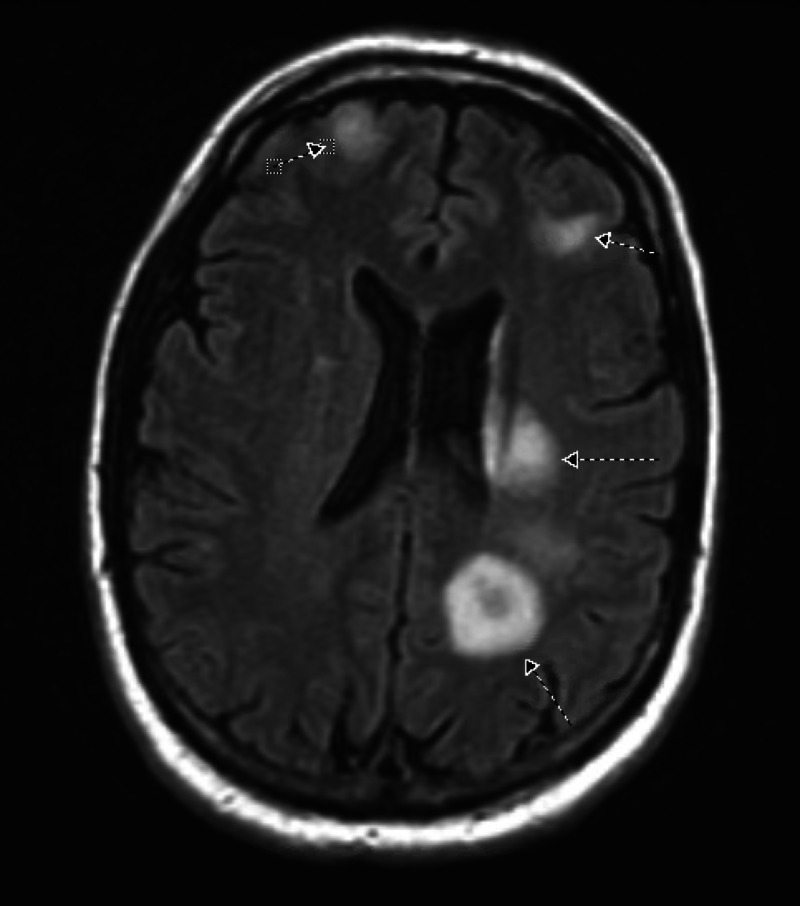
MRI of the brain Arrows indicate multiple, bilateral ring-enhancing lesions with more lesions on the left cerebrum than the right cerebrum MRI: magnetic resonance imaging

A 41-year-old male with a history of diverticulosis presented with complaints of nausea, vomiting, fever, chills, diarrhea, right shoulder pain, weakness in the right arm, and discomfort in his right leg for two weeks. At presentation, his temperature was 101 °F, his epigastric area was tender on palpation, and he had an unstable gait. The rest of his physical examination was normal, including head-eye-ear-neck-throat, respiratory system, cardiovascular system, and genitourinary system. Initial blood work was significant for a white-blood-cell count of 23,000 cells with 11% bands, alkaline phosphatase of 152 IU/L, and international normalized ratio (INR) of 1.1. CT scan of the abdomen (Figure 1) demonstrated an 8.6 x 4.3-cm heterogeneously enhancing lesion in the right lobe of the liver, a mildly hypodense 1.4-cm ovoid lesion in the right hepatic lobe medially anteriorly, multiple sigmoid diverticulosis without evidence of acute diverticulitis, and a superior right hepatic vein thrombus. He subsequently underwent a CT-guided fine-needle aspiration biopsy of the lesion, which showed a grossly purulent material, and a percutaneous drainage catheter was placed. Pathology was negative for malignant cells but showed suppurative inflammation with necrosis consistent with hepatic abscess. MRI of the brain was performed for weakness in the right arm. It showed diffusion-restricted, multiple ring-enhancing lesions with associated edema throughout the cerebrum, left > right, and the right cerebellum (Figure 2).

**Figure 3:**
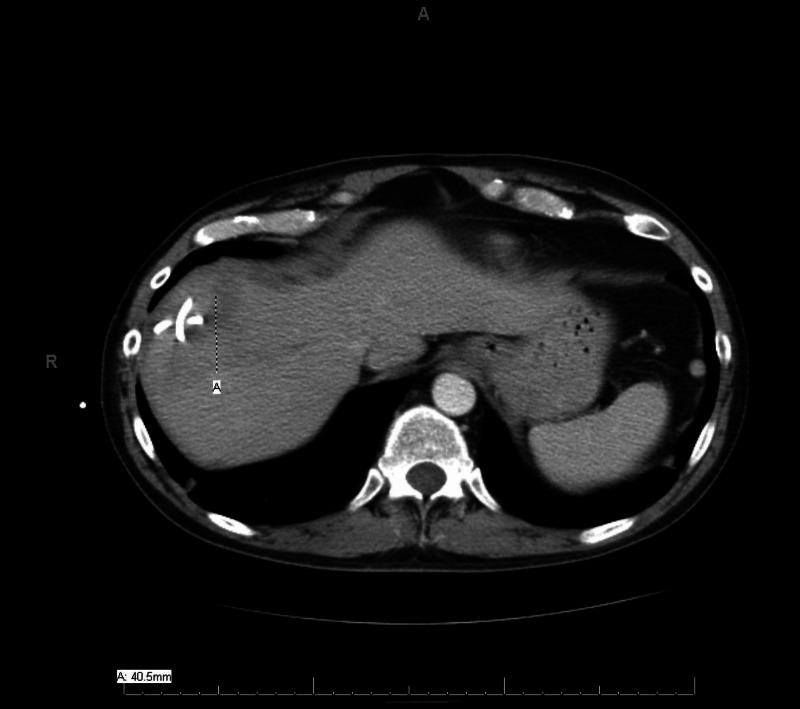
Repeat CT scan of abdomen and pelvis The cross mark indicates a significant decrease in the liver abscess size to 4.1 x 4.3 cm (repeat CT performed four weeks after the rehab treatment) CT: computed tomography

Tumor markers alpha-fetoprotein, carcinoembryonic antigen, and carbohydrate antigen (CA) 19-9 levels were normal. A working diagnosis of hepatic abscess and brain abscess was made. The patient was empirically started on vancomycin 1 g, ceftriaxone 2 g, and metronidazole 500 mg. Blood culture was positive for Fusobacterium in two bottles. Hepatic abscess cultures also returned positive for F. nucleatum. Biopsy and drainage of the brain were not done as the patient’s symptoms improved with the above treatment. His drainage catheter was removed, and he was discharged to an inpatient rehab facility where he completed a six-week course of ceftriaxone 2 g IV every 12 hours and metronidazole 500 mg every eight hours. He clinically improved, and right upper extremity weakness and shoulder pain subsided with physical and occupational therapy. Repeat CT abdomen and pelvis performed four weeks later demonstrated a significant decrease in liver abscess size to 4.1 x 4.3 cm (Figure 3) and tubular hypodensity in the superior aspect of the liver suggestive of thrombosed superior right hepatic vein branch. No anticoagulation was started for treatment of the hepatic vein thrombus, as it was likely a pylephlebitis. Due to recurrent diverticulitis, three months after his hospitalization, he had sigmoidectomy with diverting loop ileostomy, which was eventually successfully reversed. Histopathological reports from the sigmoid resection during ileostomy and the reversal surgery did not show any evidence of malignancy.

## Discussion

Fusobacterium species are common pathogens present in human flora, mainly in the oropharynx, gastrointestinal tract, and female genitalia. Fusobacterium rarely causes bacteremia, only accounting for 0.00038% of all bacteremia [1]. The majority of intra-abdominal and pelvic infections caused by F. nucleatum affect those who are >60 years of age with multiple comorbidities, including malignancies [1,3,6]. Our patient was young and did not have any comorbidities, including cancer. The source of infection in our patient was not identified for the disseminated infection. Even though the CT scan of the abdomen and pelvis was positive for diverticulosis, there was no sign of diverticulitis in the patient. Other possible sources of Fusobacterium bacteremia such as oropharynx and genitalia were also ruled out.

To the best of our knowledge, there have only been four other cases of Fusobacterium hepatic abscess associated with pylephlebitis in the international literature [4,5,7,8]. In some of the previous cases, the patients were treated with both anticoagulation and antibiotics. Our patient’s hepatic thrombosis was thought to be pylephlebitis, and he was given antibiotics only. However, the repeat CT scans of those patients who were treated without anticoagulation showed persistent portal vein thrombosis and liver atrophy [4,5]. Our patient did not have any liver atrophy on a repeat scan. The mortality rate associated with Fusobacterium species bacteremia is very low, except in cases of F. nucleatum where the mortality is as high as 30% [9].

## Conclusions

Disseminated Fusobacterium bacteremia causing multiple organ abscesses and pylephlebitis is extremely rare in young and healthy patients who carry no risk factors for the condition. Fusobacterium bacteremia usually occurs due to gastrointestinal and genitourinary infections. However, our patient did not show any obvious sources of infection.
